# Changes in Biventricular Cardiac Mechanics After Transcatheter Edge-to-Edge Repair for Severe Tricuspid Regurgitation

**DOI:** 10.1016/j.jacadv.2025.102549

**Published:** 2026-01-21

**Authors:** Giulio M. Mondellini, Antoon J.M. van den Enden, Mark M.P. van den Dorpel, Claire Ben Ren, Christiaan L. Meuwese, Isabella Kardys, Rutger-Jan Nuis, Maarten ter Horst, Marcel L. Geleijnse, Joost Daemen, Daniel Burkhoff, Nicolas M. Van Mieghem

**Affiliations:** a Department of Cardiology, Cardiovascular Institute, Erasmus University Medical Center, Rotterdam, the Netherlands; b Department of Intensive Care Adults, Erasmus University Medical Center, Rotterdam, the Netherlands; c Department of Cardiothoracic Anesthesiology, Erasmus University Medical Center, Rotterdam, the Netherlands; d Cardiovascular Research Foundation, New York City, New York, USA

**Keywords:** contractility, coupling, pressure-volume loop, right ventricle, tricuspid regurgitation

## Abstract

**Background:**

Transcatheter edge-to-edge repair (TEER) is an established therapy for severe tricuspid regurgitation (TR). Invasive pressure-volume (PV) analysis is the gold standard for characterizing ventricular function and ventricular-vascular interactions. The effects of tricuspid TEER on biventricular PV relationships are unknown.

**Objectives:**

The authors aimed to assess postprocedural changes in right (RV) and left ventricular (LV: 1) end-systolic and end-diastolic pressures and volumes; 2) ventricular-arterial coupling, expressed as end-systolic elastance (E_es_) to effective arterial elastance (E_a_) ratio; and 3) metabolic demand, represented by PV area (PVA).

**Methods:**

We used a conductance catheter to determine RV and LV PV relationships before and after tricuspid TEER. Pre- and postprocedural changes in cardiac mechanics were compared using the paired-samples *t*-test or Wilcoxon signed rank test.

**Results:**

Among the twenty-two patients (mean age 80 ± 6 years, 46% female, median LV ejection fraction of 52 [IQR 44-55]%) with severe TR, tricuspid TEER resulted in significant TR reduction and lower RV volumes (end-diastolic volume from 114.8 ± 32.2 to 102.0 ± 26.8 mL, *P* < 0.001). RV afterload increased (E_a_: 0.55 [0.47-0.81] mm Hg/mL to 0.85 [0.65-1.27] mm Hg/mL, *P* < 0.001) as did RV contractility (E_es_: from 0.46 [0.33-1.06] to 0.82 [0.55-2.07] mm Hg/mL, *P* < 0.001), with a stable RV E_es_/E_a_, preserved stroke volume, RV end-diastolic pressure, PVA, and stroke work-to-PVA ratio. LV end-diastolic volume increased, (108.0 ± 31.8-114.0 ± 32.2 mL, *P* < 0.001), whereas LV pressures, contractility, E_es_/E_a_, PVA, and stroke work-to-PVA remained unchanged.

**Conclusions:**

TR reduction with tricuspid TEER generated immediate RV volume unloading, increased RV afterload, and enhanced RV contractility, maintaining forward stroke volume and increasing LV preload.

Tricuspid regurgitation (TR) is a highly prevalent condition. Moderate or severe TR is observed in 0.6% of the general population,^1^ 3 to 5% of patients above 75 years old,^2^ nearly one-third of patients with atrial fibrillation,^3^ and more than one-third of patients with heart failure.^4^, ^5^, ^6^

Transcatheter edge-to-edge repair (TEER) has emerged as an effective technique for reducing severe TR and improving quality of life in selected patients at high risk for surgery.^7^, ^8^, ^9^

The effects of tricuspid TEER on cardiac mechanics remain poorly understood. Apart from altering intraventricular loading conditions, transcatheter valvular interventions induce changes in physiology on a biventricular level. The invasive assessment of pressure-volume (PV) relationships is the gold standard for evaluating cardiac mechanics in vivo.^10^^,^^11^

The prospective “PLUTO (Real time Pressure volume Loop monitoring as a guide for enhanced Understanding of changes in elemental cardiovascular physiology during Therapeutic strategies for hemodynamic Optimization, Cohort II: Structural Heart Interventions)” cohort-II study^12^ aims to elucidate the key modifications in cardiac energetics, ventricular loading, and ventricular-vascular coupling surrounding transcatheter valve interventions.

Deep understanding of the impact on cardiac mechanics of such interventions (ie, tricuspid TEER) is key to promptly detect patients at high-risk of hemodynamic adverse events (eg, poor ventricular-vascular coupling, low contractile reserve) and to consequently tailor their perioperative management.

Herein, we report our insights into the dynamic changes of right ventricular (RV) and left ventricular (LV) PV relationships in consecutive patients from the PLUTO-II study who underwent tricuspid TEER for symptomatic severe TR.

## Methods

### Study design

This single-center prospective observational study included patients with symptomatic severe TR who underwent tricuspid TEER at the Erasmus University Medical Center in Rotterdam, the Netherlands. The decision to proceed with tricuspid TEER was per heart team consensus.

Preprocedural assessment comprised clinical evaluation, laboratory parameters, including but not limited to N-terminal pro–B-type natriuretic peptide levels, transthoracic and transesophageal echocardiography (TEE), and right heart catheterization.

TR severity was assessed by experienced echocardiography specialists according to European Society of Cardiology guidelines, with a multiparametric approach including qualitative, semiquantitative, and quantitative echocardiography measures.^13^

Procedural success was defined as an improvement in TR of at least 1 grade based on the index TEE, which may be different from the TR as assessed by transthoracic echocardiography predischarge.

All patients consented to participate in the PLUTO-II prospective observational study registered at NCT06204783. The inclusion and exclusion criteria have been previously described.^12^ The study protocol was approved by the local medical ethics committee (Medical Ethics Committee, Erasmus University Medical Center Rotterdam, registration number: MEC-2022-0132).

### Objectives

The main study objective was to investigate the immediate changes in RV and LV PV relationships after tricuspid TEER and included: 1) changes in RV and LV end-systolic pressure (ESP) and end-diastolic pressure and volumes; 2) changes of ventricular-arterial coupling expressed by the ratio of end-systolic elastance (E_es_), (measure of the load-independent contractility) and effective arterial elastance (E_a_), (measure for afterload); and 3) changes of RV and LV metabolic demand, represented by the PV area (PVA).

### Procedural details

All tricuspid TEER procedures were performed under general anesthesia with fluoroscopic and TEE imaging guidance. Mechanical ventilation settings, use of vasoactive agents, spasmolytics, and anesthetics were per treating anesthesiologist’s discretion. Electrocardiography and invasive arterial blood pressure were monitored concomitantly. Access of the femoral vein (24-F) and common femoral artery (7-F) was obtained through an ultrasound guided puncture and Seldinger technique. A conductance catheter (CD Leycom) was consecutively introduced into the RV and LV. Apical seating of the conductance catheter was verified by fluoroscopy and visual inspection of segmental PV loops (version 3.18.1, Inca, CD Leycom). PV signals were recorded during 10 seconds in case of sinus rhythm and 15 seconds for patients in atrial fibrillation, while applying an end-expiratory hold on the mechanical ventilator.

The conductance catheter remained in the apex of the LV during the tricuspid TEER procedure. Tricuspid TEER was performed per local standard of care and the number of devices and the accepted residual TR was at the operating team’s discretion. Following leaflet approximation and stabilization, PV relationships were remeasured in the LV and the RV after repeated volume calibration (Supplemental Figure 1).

### Volume calibration

The RV and LV end-systolic volume (ESV) and end-diastolic volume (EDV) were determined by 3D-TEE (Philips EPIQ Ultrasound system, Philips Healthcare or Siemens Acuson SC2000 Prime, Siemens Healthineers AG) for volume calibration of the conductance catheter measurements, as described previously.^14^ Three-dimensional volume reconstructions were created from the 4-chamber views using full volume mode multibeat acquisition. Segmentation of 3D-TEE images into short-axis and long-axis views was performed with a dedicated, validated imaging segmentation software (3Mensio Structural Heart, Pie Medical) using a stacked model with 8-mm slices, for endocardial borders tracing, as previously described.^14^

These echocardiographic views were obtained immediately before vascular access and after device placement.

### PV relations

It is important to note that the PV acquisitions were embedded in our clinical practice. Multibeat assessments to determine RV and LV E_es_ with changing preloading conditions, through caval vein occlusions, were deemed inappropriate.

We, therefore, relied on the single-beat approach, as described by Brimioulle et al^15^^,^^16^ and Chen et al^17^ to determine RV and LV E_es_ and corresponding V_0mm Hg_.

The RV PV loop was constructed from instantaneous RV pressure and volume data smoothed with a standard low-pass filter. In order to determine the RV E_es_, the single-beat approach originally described by Brimioulle et al^15^^,^^16^ was applied. By means of PV data derived from 2 consecutive beats with similar morphology, 8 distinct pressure/time points on the ventricular pressure waveform were defined based on the first derivative of pressure change over time and its second derivative. The second derivative approach has been proposed to reduce interobserver variability in the analysis of ventricular pressure data.^18^ A sinusoidal curve was subsequently fitted through these points, using equation P = *a* + *b* · sin (*c* · *t* + *d*), where P is pressure and *t* is time, after adopting a nonlinear fit to determine coefficients a-d (Supplemental Figure 2A). P_max_ was then calculated as P_max_ = *a + 2b*. RV E_es_ represented as the slope of the ESPV relationship line, was subsequently calculated as (P_max_ ESP)/stroke volume (Supplemental Figure 2B). An example of this approach is visualized in Supplemental Figures 2A and 2B.

The single-beat algorithm comes with intrinsic limitations of mathematical assumptions and may generate unrealistic V_0mm Hg_ in patients with significant valvular disease undergoing structural heart interventions. That may affect the interpretation of the resultant E_es_ values after TEER. To overcome these limitations, we used a fixed V_0mm Hg_ (ie, the V_0mm Hg_ that was determined by means of the E_es_ before TEER), and post-TEER E_es_ was calculated as: ESP/[ESV − V_0_], where ESP is end-systolic pressure and ESV is end-systolic volume.

The comparison between the E_es_ post-TEER using the estimated V_0_ after TEER through single-beat E_es_ equation and the calculated E_es_ post-TEER using the fixed V_0_ is reported in Supplemental Material. An example of the PV reconstruction and E_es_ estimate with single-beat calculated and fixed V_0mm Hg_ are provided in Supplemental Table 1 and Supplemental Figure 3. Supplemental Table 2 illustrates the RV E_es_ values using the calculated V_0_ and the fixed V_0._

In addition to PV-based assessment of systolic function, we also measured the maximum rate of pressure change over time (dP/dt_max_). Stroke volume was estimated by the difference between RV EDV (defined as volume at the point of dP/dt_max_) and ESV. Ventricular afterload was quantified by the effective E_a_, which is the ratio of ESP/SV. Ventricular-vascular coupling was indexed by the ratio between E_es_ and E_a_. Changes in myocardial energetic demand and performance were assessed by the PVA, which represents the sum of stroke work (SW) (the area inside the PV loop) and potential energy (the area of the triangle to the left of the PV loop bounded by the end-systolic and end-diastolic PV relationships). The ratio of SW to PVA represents ventricular mechanical efficiency.^19^

### Statistical analysis

Sample size calculations for this study have been presented in detail previously.^12^ In brief, using paired *t*-tests, we estimated that 36 patients would provide 80% power at α = 0.05 to detect a mean difference of −1.779 (SD ± 3.690) in PVA. Ultimately, 22 patients were included, enabling detection of a mean difference in PVA of −2.350, as shown by post hoc power analysis.

Normality of the distributions of continuous variables was assessed by Shapiro-Wilk test. Continuous variables are expressed as mean ± SD or as median (interquartile range, IQR) if not normally distributed. Categorical data are expressed as frequencies and percentages. Pre- and postprocedural measures within the same subjects were compared using the paired-samples *t*-test or Wilcoxon signed rank test, as appropriate. Changes in continuous variables before and after tricuspid TEER were described using mean difference (before – after) with 95% CIs or median difference (before − after) with 95% CI as calculated using the Hodges-Lehmann estimator, depending on the normality of the distribution of the difference. Significance was always set at a 2-sided probability level of <0.05.

For data processing and statistical analysis, MatLab (MathWorks, R2022a) and SPSS (version 28, IBM Corp.) were used. Prism (PRISM 8.1, GraphPad Software) was used for graphs and data visualization.

## Results

Between February 2021 and June 2024, 26 patients undergoing tricuspid TEER were enrolled in the PLUTO-II study. Two patients were excluded because no tricuspid TEER was performed due to inadequate leaflet grasping. In 2 other patients, insufficient imaging quality precluded volume calibration. A flow-chart with enrolled patients, dropouts and respective causes is illustrated in Supplemental Figure 4.

Pre- and post-TEER PV loops were successfully reconstructed in 22 patients. In 2 patients, only RV-PV loops were available, and in 1 patient, only LV-PV loops. The mean age was 80.4 ± 5.7 years, 54% were male and mean TRI-SCORE^20^ was 6 ± 2. Seven (31.8%) patients had a cardiac implantable electronic device. Patients presented in NYHA functional class III (54%) or IV (46%). The median N-terminal pro–B-type natriuretic peptide was 248 [IQR 106, 426] pmol/L, and the mean creatinine-based estimated glomerular filtration rate was 45 ± 18 mL/min/1.73 m^2^. TR was severe in 10 patients, massive in 10 patients, and torrential in 2 patients. In 14 patients (63%), the etiology was atrial secondary TR (A-STR), 2 patients had mixed A-STR and degenerative TR, 3 had ventricular secondary TR, and 3 exhibited overlapping features of both ventricular secondary TR and A-STR. The median LV ejection fraction was 52% [IQR 44, 55%], the mean baseline tricuspid annular plane systolic excursion (TAPSE) was 17± 5 mm. Further details of patient demographics are summarized in Table 1. Baseline right heart catheterization showed a mean pulmonary artery (PA) pressure of 19 ± 5 mm Hg with mean capillary wedge pressure of 10 ± 4 mm Hg. The mean cardiac output, measured by thermodilution, was 3.4 ± 1.0 L/min, and pulmonary vascular resistance was 243 ± 126 dyn × s/cm^5^. Eleven patients (50%) exhibited a mean PA pressure above 20 mm Hg, due left-sided heart disease (type 2).

**Table 1:** Demographics and Clinical Characteristics (N = 22)

Demographics	
Age, y	80.4 ± 5.67
Female, n%	10 (46%)
BMI, kg/m^2^	24.73 ± 4.81
NYHA functional class, n (%)	
III	12 (54)
IV	10 (46)
TRI-SCORE	6.0 ± 2.08
Prior medical history	
Arterial hypertension, n (%)	14 (63.6)
Chronic kidney disease^a^, n (%)	13 (59.1)
Diabetes mellitus, n (%)	6 (27.3)
Atrial fibrillation, n (%)	21 (95.5)
Stroke or TIA, n (%)	3 (13.6)
Prior myocardial infarction, n (%)	3 (13.6)
Coronary artery disease, n (%)	11 (50.0)
Pacemaker lead, n (%)	7 (31.8)
COPD, n (%)	3 (13.6)
Malignancy, n (%)	6 (27.3)
Laboratory	
Hemoglobin, mmol/L	7.97 ± 0.88
Creatinine, μmol/L	134.09 ± 53.36
e-GFR (ml/min/1.73 m^2^)^b^	45.32 ± 17.60
NT-proBNP, pmol/L	248 (106, 426)
C-reactive protein, mg/L	2.3 (1.1, 8.1)
White blood cell count, x 10^9^/L	6.39 ± 1.38
Baseline echocardiography	
Tricuspid regurgitation severity, n (%)	
Severe	10 (46)
Massive or torrential	12 (54)
Tricuspid regurgitation etiology, n (%)	
Secondary TR	20 (90.2)
Atrial	14 (63)
Ventricular	3 (13.6)
Overlapping features, A-STR - V-STR	3 (13.6)
Mixed primary – secondary TR	2 (9.8)
LVEF (%)	52 (44, 55)
TAPSE, mm	17 ± 5
Baseline right heart catheterization, n = 21	
Systolic PA pressure, mm Hg	30 ± 7
Diastolic PA pressure, mm Hg	13 ± 4
Mean PA pressure, mm Hg	19 ± 5
Pulmonary capillary wedge pressure, mm Hg	10 ± 4
Pulmonary vascular resistance, dynes × sec/cm^5^	243 ± 126
Cardiac output, L/min	3.4 ± 1.0
Postprocedural echocardiography	
Tricuspid regurgitation severity, n%	
Mild	4 (18.2)
Mild-moderate	3 (13.4)
Moderate	8 (36.4)
Moderate-severe	4 (18.2)
Severe	3 (13.4)
Mean atrioventricular PG, mm Hg	2 (1,3.75)

Continuous variables are presented as mean ± SD or median (25th, 75th percentile).

A-STR = atrial secondary TR; BMI = body mass index; COPD = chronic obstructive pulmonary disease; e-GFR = estimated glomerular filtration rate; LVEF = left ventricular ejection fraction; NT-proBNP = N-terminal pro–B-type natriuretic peptide; PA = pulmonary artery; PG = pressure gradient; TAPSE = tricuspid annular plane systolic excursion; TIA = transient ischemic attack; TR = tricuspid regurgitation; V-STR = ventricular secondary TR.

a Chronic Kidney Disease was graded according to the 2015 Kidney Disease Improving Global Outcomes definition.

b e-GFR was calculated according to Chronic Kidney Disease Epidemiology Collaboration principles.

Tricuspid TEER was performed successfully in all patients, with appropriate devices deployment to reach satisfactory intraoperatively TR reduction per operator`s discretion, without elevated mean transvalvular gradient. Tricuspid TEER was accomplished with TriClip (Abbott) and Pascal ACE (Edwards Lifesciences) devices, with a median number of 2 devices per patient. At predischarge ultrasound assessment, residual TR was ≤ moderate in 15 patients and > moderate in 7 patients. Intraprocedural success, according to Tricuspid Valve Academic Research Consortium, was achieved in 15 patients. Intraprocedural success was not met in the remaining 7 patients because of more than moderate residual TR.

At 30 days follow-up, all patients were alive, 2 were scheduled for redo tricuspid TEER due to single leaflet device attachment, one patient received a permanent pacemaker implantation for atrioventricular block, one experienced ischemic stroke, and one suffered a major access site bleeding. After a median follow-up time of 474 [IQR 175, 597] days, 7 patients (31.8%) died, 5 of whom exhibited more than moderate residual TR.

**Figure 1:**
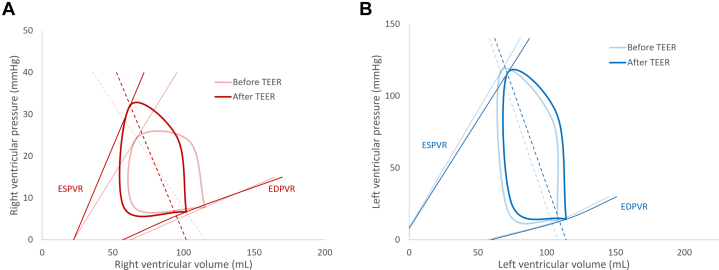
**Pressure-Volume Loops Before and After Tricuspid Transcatheter Edge-to-Edge Repair** Average right (A) and left ventricle (B) pressure-volume loops, before and after tricuspid transcatheter edge-to-edge repair. These pressure-volume reconstructions appraise the immediate changes in cardiac mechanics induced by transcatheter edge-to-edge repair for severe tricuspid regurgitation, based on conductance catheter measurements in 22 patients. Ventricular contractile properties are described by the end-systolic pressure-volume relationship, and its slope (known as end-systolic elastance). The diastolic function is described by the end-diastolic pressure-volume relationship. Afterload can be characterized by the effective arterial elastance (dashed lines). After transcatheter edge-to-edge repair, right ventricular end-diastolic volume decreased, while right ventricular arterial elastance and right ventricular end-systolic elastance increased. In the left ventricle, end-diastolic volume increased with stable left ventricular end-diastolic pressure and nonsignificant changes in left ventricular end-systolic elastance and left ventricular arterial elastance, immediately after the procedure. EDPVR = end-diastolic pressure-volume relationship; ESPVR = end-systolic pressure-volume relationship; TEER = transcatheter edge-to-edge repair.

Changes in RV and LV cardiac mechanics after tricuspid TEER are summarized in Table 2, and the aggregate changes in the PV relationship are illustrated in Figure 1.

**Table 2:** The Change in RV and LV Cardiac Mechanics Induced by Tricuspid TEER

	RV Before TEER (n = 21)	RV After TEER (n = 21)	*P* Value	Mean Difference (95% CI)	LV Before TEER (n = 20)	LV aAfter TEER (n = 20)	*P* Value	Mean Difference (95% CI)
Heart rate, beats/min	70 ± 15	62 ± 12	0.003	−7.94 (−12.75 to −3.13)	64 ± 14	61 ± 14	0.182	−3.02 (−7.57 to 1.54)
End-systolic pressure, mm Hg	24.4 ± 8.8	32.2 ± 14.4	<0.001	7.79 (4.04-11.54)	116.8 ± 21.0	115.6 ± 19.5	0.771	−1.18 (−9.52 to 7.17)
End-diastolic pressure, mm Hg	7.8 ± 3.8	6.7 ± 4.0	0.153	−1.07 (−2.57 to 0.43)	13.4 ± 3.7	14.3 ± 4.7	0.255	0.88 (−0.69 to 2.45)
End-systolic volume, mL	66.8 ± 19.3	62.3 ± 19.7	0.005	−4.52 (−7.44 to −1.59)	66.5 ± 25.4	71.3 ± 25.6	0.007	4.79 (1.44-8.14)
End-diastolic volume, mL	114.8 ± 32.2	102.0 ± 26.8	<0.001	−12.80 (−17.53 to −8.07)	108.0 ± 31.8	114.0 ± 32.2	0.001	5.98 (2.86-9.10)
Stroke volume, mL	42.9 ± 18.2	37.4 ± 15.7	0.133	−5.53 (−12.91 to 1.85)	38.3 ± 9.9	38.6 ± 13.5	0.880	0.36 (−4.53 to 5.24)
dP/dt _max_, mm Hg/s	190.9 ± 88.5	233.4 ± 94.0	0.002	42.50 (13.82-71.18)	1,033.7 ± 224.8	957.9 ± 215.8	0.012	−75.80 (−132.63 to −18.96)
E_es_, mm Hg/mL	0.46 (0.33-1.06)	0.82 (0.55-2.07)	<0.001	0.66 (0.26-1.07)	1.63 (1.09-2.25)	1.53 (1.06-2.33)	0.601	−0.02 (−0.21 to 0.17)
V_120_, mL	242.0 (153.2-391.8)	159.9 (127.6-194.3)	0.001	−119.37 (−179.60 to −59.14)	80.5 (58.2-90.7)	83.1 (63.6-95.5)	0.056	8.34 (−0.22 to 16.89)
E_a_, mm Hg/mL	0.55 (0.47-0.81)	0.85 (0.65-1.27)	<0.001	0.36 (0.16-0.56)	3.29 (2.12-4.09)	3.13 (2.35-4.33)	0.765	0.12 (−0.41 to 0.65)
E_es_/E_a_	0.98 (0.64-1.73)	1.30 (0.59-2.22)	0.054	0.52 (−0.04 to 1.07)	0.50 (0.42-0.62)	0.52 (0.36-0.64)	0.926	0.02 (−0.11 to 0.15)
Stroke work, mm Hg/mL	895.3 (529.3-1,166.3)	866.4 (605.0-1,556.7)	0.065	201.93 (−53.18 to 457.03)	3,436.6 (2,772.4-4,535.8)	4,243.1 (2,962.6-5,035.5)	0.403	249.38 (−361.30 to 860.07)
Potential energy, mm Hg/mL	514.0 (210.5-799.6)	352.5 (159.1-925.7)	0.931	−72.61 (−252.68 to 107.46)	3,778.6 (2,869.7-5,431.9)	4,276.1 (3,107.2-5,179.9)	0.550	−185.09 (−1,082.90 to 712.72)
Pressure-volume Area, mm Hg/mL	1,458.0 (805.3-1997.9)	1,322.6 (752.2-2,457.8)	0.149	226.25 (−139.32 to 591.83)	7,663.6 (6,501.5-9,996.3)	8,363.0 (7,067.3-10,193.4)	0.881	14.33 (−1,174.68 to 1,203.34)
SW/PVA ratio	0.624 ± 0.15	0.671 ± 0.17	0.118	0.047 (−0.030 to 0.124)	0.478 ± 0.10	0.487 ± 0.13	0.845	0.009 (−0.061 to 0.078)
Ejection time, s	0.256 ± 0.043	0.289 ± 0.047	0.005	0.033 (0.013-0.054)	0.287 ± 0.044	0.313 ± 0.073	0.007	0.026 (0.008-0.045)
dP/dV, mm Hg/mL	23.44 (15.19-30.36)	20.7 (11.19-25.45)	0.089	−3.39 (−8.00 to 1.23)	40.86 (30.95-48.81)	44.04 (31.75-54.68)	0.560	3.86 (−2.43 to 10.14)
-dP/dt min, mm Hg/s	−165.0 (−195.0 to −131.0)	−250.3 (−354.8 to 186.6)	<0.001	−80.18 (-136.54 to −23.82)	−1,046.6 (−1,241.1 to −907.0)	−1,008.4 (−1,188.4 to −923.4)	0.409	37.90 (−45.75 to 106.55)^a^
Tau, ms	56.0 (42.3-108.3)	42.4 (32.5-53.7)	0.007	−20.10 (−86.20 to −4.70)^a^	38.5 (36.0-44.0)	40.55 (36.6-45.0)	0.147	2.02 (−0.78 to 4.81)

Variables are presented as mean ± SD or median (25th, 75th percentile) according to normality of the distribution.

dP/dt_max_ = maximum rate of pressure change over time; E_a_ = arterial elastance; E_es_ = end-systolic elastance; LV = left ventricular; RV = right ventricular; PVA = pressure-volume area; SW = stroke work; TEER = transcatheter edge-to-edge repair; V = volume.

a Median difference and 95% CI, calculated using the Hodges-Lehmann estimator.

### RV PV assessments

**Figure 2:**
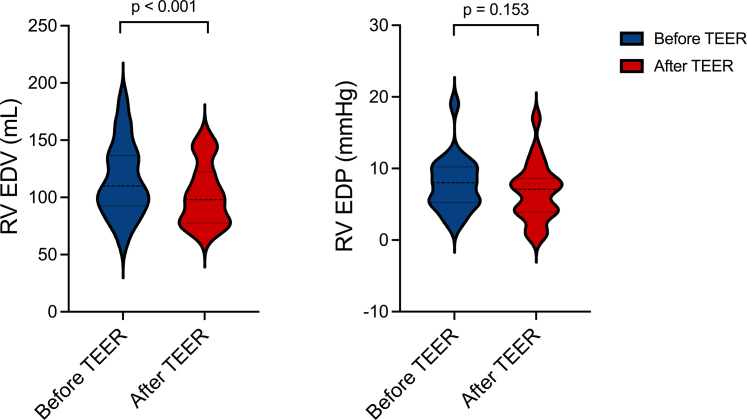
**Right Ventricular End-Diastolic Volume and Pressure Changes** Violin plots representing right ventricular end-diastolic volume (left panel) and pressure (right panel) values before and after tricuspid transcatheter edge-to-edge repair. EDP = end-diastolic pressure; EDV = end-diastolic volume; RV = right ventricular; TEER = Transcatheter edge-to-edge repair.

**Figure 3:**
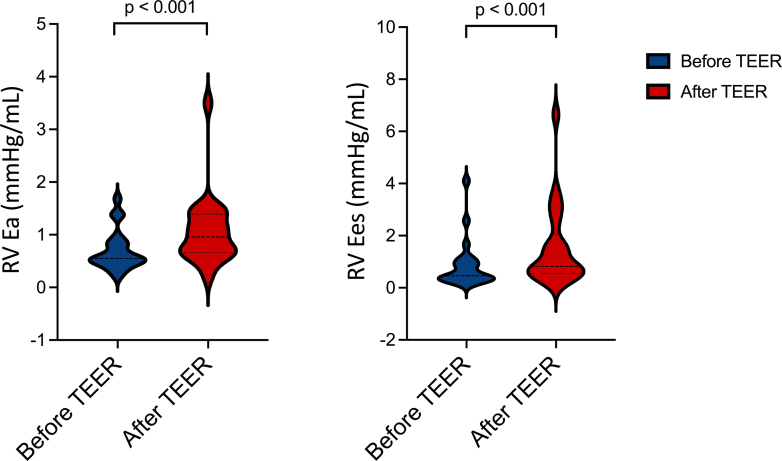
**Right Ventricular Effective Arterial Elastance and End-Systolic Elastance Changes** Violin plots representing right ventricular effective arterial elastance (left panel) and end-systolic elastance (right panel) values before and after tricuspid transcatheter edge-to-edge repair. E_a_ = effective arterial elastance; E_es_ = end-systolic elastance; RV = right ventricular; TEER = transcatheter edge-to-edge repair.

Tricuspid TEER resulted in immediate reductions in RV EDV (from 114.8 ± 32.2 to 102.0 ± 26.8 mL, <0.001). RV ESP increased (24.4 ± 8.8-32.2 ± 14.4 mm Hg, *P* < 0.001), while end-diastolic pressure remained unchanged (7.8 ± 3.8-6.7 ± 4.0 mm Hg, *P* = 0.153) (Figure 2). RV E_a_ increased following TEER from 0.55 [IQR 0.47, 0.81] to 0.85 [IQR 0.65, 1.27] mm Hg/mL (*P* < 0.001) (Figure 3, left panel). RV contractility, indexed by E_es_, increased following TEER from 0.46 [IQR 0.33, 1.06] to 0.82 [IQR 0.55, 2.07] mm Hg/mL (*P* < 0.001; Figure 3, right panel); dP/dt_max_ also increased from 190.9 ± 88.5 to 233.4 ± 94.0 mm Hg/s after TEER (*P* = 0.002). RV-PA coupling indexed by RV E_es_/E_a_ trended to increase from 0.98 [IQR 0.64, 1.73] to 1.30 [IQR 0.59, 2.22] (*P* = 0.054). Additional visual representation of changes in RV contractility, indexed by E_es_, with a paired dot plot is illustrated in Supplemental Figure 5.

Notably, RV E_a_ and RV E_es_ increased after tricuspid TEER in 18 out of the 21 patients with available RV loops. Three of the 7 patients with greater than moderate residual TR, had no increase in RV E_a_ and RV E_es_. An increase in RV E_a_ and E_es_ was observed in all patients with ≤ moderate residual TR and available RV PV loops. In 6 of 8 patients with impaired RV E_es_/E_a_ at baseline (ie < 0.8), RV E_a_ and E_es_ increased after tricuspid TEER, resulting in an RV E_es_/E_a_ ratio >0.8 in 4 patients.

RV stroke volume did not change significantly (from 42.9 ± 18.2-37.4 ± 15.7, *P* = 0.133, Table 2), suggesting that forward flow increased due to the diminished regurgitant volume following tricuspid TEER. The median RV PVA (1,458.0 [IQR 805.3, 1997.9] mm Hg/mL vs 1,322.6 [IQR 752.2, 2,457.9]) mm Hg/mL, *P* = 0.149) and SW/PVA ratio (0.62 ± 0.15 vs 0.67 ± 0.17, *P* = 0.118) did not change significantly following TEER. The median 3D RV ejection fraction and TAPSE remained stable (RVEF pre 44% [IQR 31, 49] and post 42% [IQR 29, 50] %, *P* = 0.889, mean TAPSE pre 17 ± 5 mm and post 18 ± 5 mm, *P* = 0.834).

### LV PV assessments

**Figure 4:**
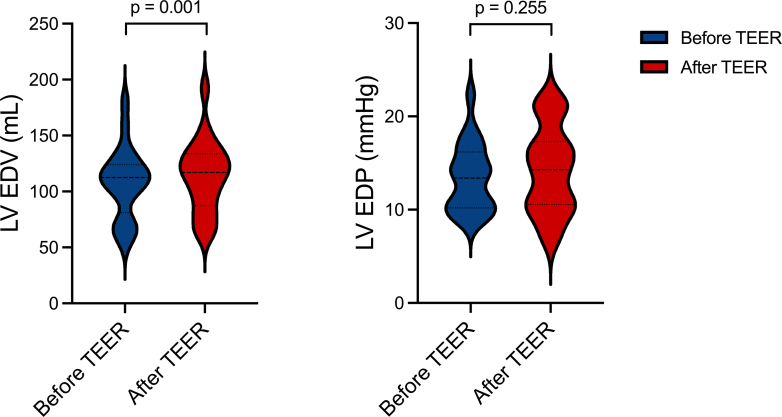
**Left Ventricular End-Diastolic Volume and Pressure Changes** Violin plots representing left ventricular end-diastolic volume (left panel) and pressure (right panel) values before and after tricuspid transcatheter edge-to-edge repair. EDP = end-diastolic pressure; EDV = end-diastolic volume; LV = left ventricular; TEER = transcatheter edge-to-edge repair.

LV EDV mildly increased (108.0 ± 31.8-114.0 ± 32.2 mL, *P* < 0.001), while LV ESP remained stable after tricuspid TEER (13.4 ± 3.7 vs 14.3 ± 4.7 mm Hg, *P* = 0.255) (Figure 4).

There were nonsignificant changes in LV E_es_ and LV E_a_ immediately after tricuspid TEER. LV stroke volume, LV aortic coupling (E_es_/E_a_ ratio), PVA, and SW/PVA did not change (Table 2).

Three of the 5 patients with greater than moderate residual TR and no meaningful increases in RV E_a_ and E_es_ did not show increased LV EDVs after tricuspid TEER.

Examples of biventricular PV loops before and after tricuspid TEER in 2 patients with optimal TR reduction are illustrated in Supplemental Figures 6 and 7.

## Discussion

We evaluated the immediate effects of tricuspid TEER on RV and LV PV relationships in patients with symptomatic severe TR. We showed that tricuspid TEER was associated with: 1) reduced RV EDV; 2) increased total RV afterload; 3) increased RV contractility; and 4) increased LV EDV (Central Illustration).

**Central Illustration:**
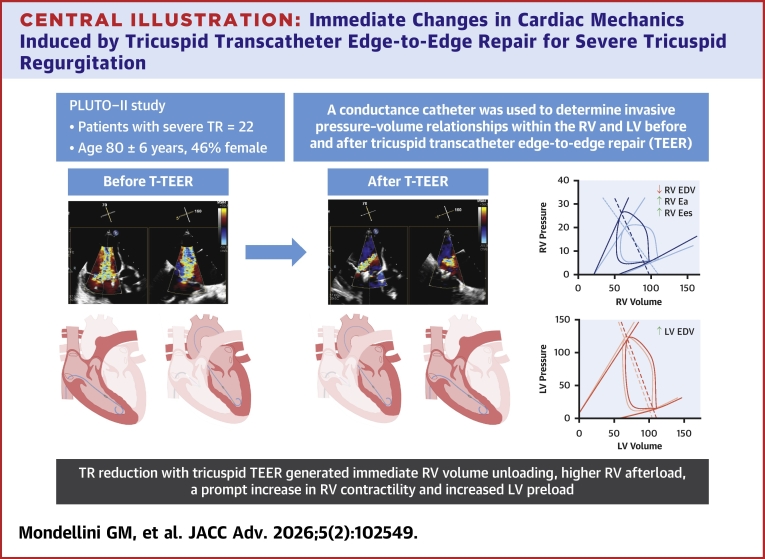
**Immediate Changes in Cardiac Mechanics Induced by Tricuspid Transcatheter Edge-to-Edge Repair for Severe Tricuspid Regurgitation** E_a_ = effective arterial elastance; E_es_ = end-systolic elastance; EDV = end-diastolic volume; LV = left ventricular; PLUTO = Real-time Pressure volume Loop monitoring as a guide for enhanced Understanding of changes in elemental cardiovascular physiology during Therapeutic strategies for hemodynamic Optimization; RV = right ventricular; T-TEER = tricuspid transcatheter edge-to-edge repair; TR = tricuspid regurgitation.

Tricuspid TEER has become a valuable catheter-based technique to mitigate TR with favorable effects on quality of life.^7^, ^8^, ^9^ The immediate effects of successful tricuspid TEER on RV and LV cardiac mechanics remain unknown.

We demonstrated that the reduction in regurgitant volume after tricuspid TEER reduces RV EDV and increases RV afterload as reflected by the elevated E_a_, which is a lumped parameter of downstream pressure and intrinsic properties of the ventricle and pulmonary vasculature.^10^ RV ESP after TEER increased by approximately 8 mm Hg as compared to baseline, which is consistent with the 7 mm Hg increase in PA systolic pressure after transcatheter tricuspid valve replacement in a recent study including 20 patients with severe TR.^21^

It was consistently observed that RV E_es_ and dP/dt_max_ increased following TEER, which indicates incremental RV contractility. Changes in right coronary perfusion, pharmacologic agents, mechanical ventilation settings, and sympathetic or parasympathetic activation cannot explain the change in RV contractility in our cohort. This phenomenon may reflect the Anrep effect, which is a so-called “homeometric” autoregulation mechanism^22^ that is independent of preload or heart rate variations and preserves stroke volume and cardiac output in response to acutely increased afterload.^23^

The Anrep effect leads to the recruitment of dormant myosin heads that transition into a contraction-ready state for actin-myosin binding to form force-generating cross-bridges to increase contractility.^24^^,^^25^ While the mechanisms underlying the Anrep effect are not fully understood, new insights were provided from an experimental model of hypertrophic obstructive cardiomyopathy^26^ in which LV outflow tract obstruction was associated with a pathological and persistent enhancement of LV contractility, that was reversed with the decay of LV afterload after percutaneous transluminal septal myocardial ablation.

We observed this Anrep effect in 18/21 patients. The 3 patients with no evidence of Anrep effect had > moderate residual TR which suggests that increased afterload achieved by effective TR reduction is required to generate this phenomenon and underscores the importance of targeting ≤ moderate residual TR after TEER.

Alternative explanations for the observed increase in RV Ees may include conformational changes in RV geometry following leaflet approximation and restoration of septal mechanics. These changes, likely resulting from reduced RV volume overload, may contribute to a more efficient RV ejection pattern.

Importantly, no patient experienced RV decompensation or low cardiac output syndrome following TEER despite marked reduction of TR. Low cardiac output state after transcatheter tricuspid interventions is rare and reported in only 2.8% of patients in the multicenter TriValve registry,^27^ 0.6% in the TRILUMINATE (The Trial to Evaluate Cardiovascular Outcomes in Patients Treated with the Tricuspid Valve Repair System Pivotal) randomized trial,^7^ and 1% in the TRISCEND-II (EVOQUE Transcatheter Tricuspid Valve Replacement: Pivotal Clinical Investigation of Safety and Clinical Efficacy Using a Novel Device) pivotal trial.^28^ We hypothesize that the increase in RV contractility after successful tricuspid repair or replacement may explain this low occurrence of acute RV failure. This may be particularly relevant in the heart failure population, where TR is relatively common^29^^,^^30^ and in which the heart’s ability to effectively increase output in response to changes in preload and afterload is blunted.^31^

We observed reduced RV EDV and increased forward flow following tricuspid TEER, resulting in higher LV EDVs. This is consistent with Kresoja et al,^32^ who described the role of ventricular interdependence after tricuspid TEER with cardiac magnetic resonance imaging, showing alleviation of diastolic leftward shift of the septum and increased LV EDV.

Notably, the LV tolerated the increased preload after tricuspid TEER with stable LV end-diastolic pressure, E_es_, E_es_/E_a_ ratio (i.e., LV-aortic coupling), and SW/PVA ratio.

In terms of ventricular remodeling, our data expand the knowledge on immediate changes after tricuspid TEER. A recent echocardiographic study reported early RV volume unloading (reduction of RV EDV) and subsequent RV structural remodeling after transcatheter tricuspid valve replacement.^33^ Importantly, the remodeling process was not completed within the first months but progressed up to 3 years after the intervention.

### Study limitations

Our study has the intrinsic limitations of a single-center mechanistic investigation and our findings should be interpreted in the context of the study’s exploratory design and limited sample size, which may restrict generalizability. Still, this cohort represents the largest clinical experience in which RV and LV PV relationships were evaluated in the context of structural heart interventions. We used single-beat algorithms, which come with intrinsic limitations of mathematical assumptions, to determine E_es_ and corresponding parameters reflecting the myocardial metabolic demand which have never been formally validated in vivo or with tricuspid valve interventions. All patients were treated under general anesthesia and the use of sedatives and vasoactive substances may represent uncontrollable confounders that should be considered when interpreting our observations. We were not able to assess invasive PV relationships beyond the intraoperative environment, characterized by the presence of factors such as by mechanical ventilation anesthethics and vasoactive agents. Although pre- and post-TEER acquisitions were performed under similar conditions in terms of mechanical ventilation, anesthetics and vasoactive drug dosages, we cannot exclude the cumulative effect of these agents and circumstances. We did not detect any changes in LV cardiac output after tricuspid TEER. While ventricular PV changes were obtained immediately after the intervention, we hypothesize that more time may be required for the LV to experience meaningful improvement after TR reduction (eg weeks/months). For the E_es_ post-TEER, we used the fixed V_0_ method to enhance the interpretability of the acquired PV loops. In Supplementary Table 1, we demonstrate no difference in RV E_es_ using the single-beat V_0_ vs the fixed V_0_.

We relied on 3D TEE imaging for volume calibration rather than on thermodilution and hypertonic saline boluses. We previously demonstrated that echocardiography can be used as an alternative to invasive hypertonic saline and thermodilution for volume calibration in invasive PV studies.^14^ While convenient and practical, it is acknowledged that 3D-TEE may underestimate ventricular volumes as compared to other modalities such as magnetic resonance.^34^^,^^35^ However, cardiac magnetic resonance imaging poses logistical challenges and excludes immediate real-time assessment of biventricular mechanics in the catheterization laboratory, with a patient under anesthesia and mechanical ventilation. Additionally, 3D-TEE reconstruction was not validated against cardiac magnetic resonance values, which remains the gold standard for volumetry assessment. Nevertheless, rather than looking at the absolute numbers and values (of volume), we believe the trends and direction of volume changes (before vs after tricuspid TEER) are relevant for our study. It is also important to note that reverse remodeling may take time to occur and we only reported on procedural and predischarge data.

TR was assessed combining qualitative, semiquantitative, and quantitative measures. In many cases, full quantitative evaluation was not feasible (ie, multiple or irregular jets) mainly due to missing regurgitant volume and effective regurgitant orifice area. Nevertheless, TR was assessed by experienced imaging cardiologists with a multiparametric approach. Our findings only apply to the selected patient population in this cohort. For example, patients with severe RV dysfunction or severe RV to PA uncoupling were underrepresented. Nevertheless, this study is the first to describe the RV response following transcatheter tricuspid valve repair.

Procedural success was based on TEE assessment at the end of the index procedure, which may be different from the transthoracic echocardiography assessment predischarge that determined the reported residual TR and may explain why the 30% rate of patients with > moderate TR is somewhat higher than what is reported in prospective studies that included a highly selected patient population with severe TR.

More research is required to study these effects in larger sets of different TR phenotypes (eg primary, atrial secondary, ventricular secondary, cardiac implantable electronic device–related) and with different repair and replacement technologies. Furthermore larger studies with longer follow-up time are required to evaluate biventricular remodeling and its long term prognostic impact.

## Conclusions

TR reduction with tricuspid TEER in patients with severe TR generated immediate RV volume unloading and higher RV afterload. The prompt increase in RV contractility maintained forward stroke volume and increased LV preload after tricuspid TEER.

## Funding support and author disclosures

Dr Van Mieghem has received research grant support from 10.13039/100011949Abbott Vascular, 10.13039/100008497Boston Scientific, 10.13039/100004374Medtronic, 10.13039/501100002973Daiichi Sankyo, 10.13039/100024655Teleflex, PulseCath BV; and advisory fees from Anteris, JenaValve, Amgen, Abbott Vascular, Boston Scientific, Medtronic, Daiichi Sankyo, Teleflex, PulseCath BV. Dr Daemen received institutional grant/research support from Abbott Vascular, Boston Scientific, 10.13039/100030776ACIST Medical, Medtronic, Pie Medical, and ReCor medical; and consultancy and speaker fees from Abbott Vascular, Abiomed, ACIST medical, Boston Scientific, Cardialysis BV, CardiacBooster, Kaminari Medical, ReCor Medical, PulseCath, Pie Medical, Sanofi, Siemens Health Care, and Medtronic. DrNuis received research grant support from 10.13039/501100006484Vifor Pharma, Merill; and consulting fees from Edwards Lifesciences, Abbott, and Boston Scientific. Dr Meuwese has received research grant support from the Dutch Heart Foundation and Fonds SGS; advisory fees for Getinge; speaker fees from Abbott and AOP Medical. Dr van den Enden has received personal fees from Abiomed and Angiodynamics. All other authors have reported that they have no relationships relevant to the contents of this paper to disclose.

## References

[1] Topilsky Y., Maltais S., Medina Inojosa J. (2019). Burden of tricuspid regurgitation in patients diagnosed in the community setting. JACC Cardiovasc Imaging.

[2] Singh J.P., Evans J.C., Levy D. (1999). Prevalence and clinical determinants of mitral, tricuspid, and aortic regurgitation (the Framingham Heart Study). Am J Cardiol.

[3] Patlolla S.H., Schaff H.V., Nishimura R.A. (2022). Incidence and burden of tricuspid regurgitation in patients with atrial fibrillation. J Am Coll Cardiol.

[4] Heitzinger G., Pavo N., Koschatko S. (2023). Contemporary insights into the epidemiology, impact and treatment of secondary tricuspid regurgitation across the heart failure spectrum. Eur J Heart Fail.

[5] Messika-Zeitoun D., Verta P., Gregson J. (2020). Impact of tricuspid regurgitation on survival in patients with heart failure: a large electronic health record patient-level database analysis. Eur J Heart Fail.

[6] Bartko P.E., Hulsmann M., Hung J. (2020). Secondary valve regurgitation in patients with heart failure with preserved ejection fraction, heart failure with mid-range ejection fraction, and heart failure with reduced ejection fraction. Eur Heart J.

[7] Sorajja P., Whisenant B., Hamid N. (2023). Transcatheter repair for patients with tricuspid regurgitation. N Engl J Med.

[8] Lurz P., Rommel K.P., Schmitz T. (2024). Real-world 1-Year results of tricuspid edge-to-edge repair from the bRIGHT study. J Am Coll Cardiol.

[9] Donal E., Dreyfus J., Leurent G. (2025). Transcatheter edge-to-edge repair for severe isolated tricuspid regurgitation: the Tri.Fr randomized clinical trial. JAMA.

[10] Brener M.I., Masoumi A., Ng V.G. (2022). Invasive right ventricular pressure-volume analysis: basic principles, clinical applications, and practical recommendations. Circ Heart Fail.

[11] Bastos M.B., Burkhoff D., Maly J. (2020). Invasive left ventricle pressure-volume analysis: overview and practical clinical implications. Eur Heart J.

[12] van den Enden A.J.M., van den Dorpel M.M.P., Bastos M.B. (2022). Invasive real time biventricular pressure-volume loops to monitor dynamic changes in cardiac mechanoenergetics during structural heart interventions: design and rationale of a prospective single-center study. Struct Heart.

[13] Vahanian A., Beyersdorf F., Praz F. (2022). 2021 ESC/EACTS guidelines for the management of valvular heart disease. Eur Heart J.

[14] van den Dorpel M.M.P., van den Enden A.J.M., Verhemel S. (2024). Validation of volume calibration by echocardiography for invasive ventricular pressure volume studies in transcatheter valve interventions. Struct Heart.

[15] Brimioulle S., Wauthy P., Ewalenko P. (2003). Single-beat estimation of right ventricular end-systolic pressure-volume relationship. Am J Physiol Heart Circ Physiol.

[16] Richter M.J., Peters D., Ghofrani H.A. (2020). Evaluation and prognostic relevance of right ventricular-arterial coupling in pulmonary hypertension. Am J Respir Crit Care Med.

[17] Chen C.H., Fetics B., Nevo E. (2001). Noninvasive single-beat determination of left ventricular end-systolic elastance in humans. J Am Coll Cardiol.

[18] Bellofiore A., Vanderpool R., Brewis M.J., Peacock A.J., Chesler N.C. (2018). A novel single-beat approach to assess right ventricular systolic function. J Appl Physiol.

[19] Suga H. (1990). Ventricular energetics. Physiol Rev.

[20] Dreyfus J., Audureau E., Bohbot Y. (2022). TRI-SCORE: a new risk score for in-hospital mortality prediction after isolated tricuspid valve surgery. Eur Heart J.

[21] Angellotti D., Bartkowiak J., Samim D. (2025). Acute haemodynamic changes following transcatheter tricuspid valve replacement. Eur J Heart Fail.

[22] Sarnoff S.J., Mitchell J.H., Gilmore J.P., Remensnyder J.P. (1960). Homeometric autoregulation in the heart. Circ Res.

[23] Meinert-Krause J.P., Mechelinck M., Hein M., Habigt M.A. (2024). Intrinsic mechanisms of right ventricular autoregulation. Sci Rep.

[24] Reil J.C., Reil G.H., Kovacs A. (2020). CaMKII activity contributes to homeometric autoregulation of the heart: a novel mechanism for the anrep effect. J Physiol.

[25] Nyitrai M., Geeves M.A. (2004). Adenosine diphosphate and strain sensitivity in myosin motors. Philos Trans R Soc Lond B Biol Sci.

[26] Sequeira V., Maack C., Reil G.H., Reil J.C. (2024). Exploring the connection between relaxed myosin states and the anrep effect. Circ Res.

[27] Rommel K.P., Taramasso M., Ludwig S. (2023). Low-cardiac output syndrome after tricuspid valve repair: insights from the TriValve registry. JACC Cardiovasc Interv.

[28] Hahn R.T., Makkar R., Thourani V.H. (2025). Transcatheter valve replacement in severe tricuspid regurgitation. N Engl J Med.

[29] Hahn R.T., Lindenfeld J., Bohm M. (2024). Tricuspid regurgitation in patients with heart failure and preserved ejection fraction: JACC state-of-the-art review. J Am Coll Cardiol.

[30] Mondellini G.M., Verbrugge F.H., Guazzi M. (2023). Transcatheter repair for patients with tricuspid regurgitation. N Engl J Med.

[31] Verbrugge F.H., Guazzi M., Testani J.M., Borlaug B.A. (2020). Altered hemodynamics and end-organ damage in heart failure: impact on the lung and kidney. Circulation.

[32] Kresoja K.P., Rosch S., Schober A.R. (2024). Implications of tricuspid regurgitation and right ventricular volume overload in patients with heart failure with preserved ejection fraction. Eur J Heart Fail.

[33] Weckbach L.T., Kothieringer M., Stolz L. (2025). Long-term right ventricular reverse remodeling and functional improvement following transcatheter tricuspid valve replacement. JACC Cardiovasc Interv.

[34] Gopal A.S., Chukwu E.O., Iwuchukwu C.J. (2007). Normal values of right ventricular size and function by real-time 3-dimensional echocardiography: comparison with cardiac magnetic resonance imaging. J Am Soc Echocardiogr.

[35] Soliman O.I., Krenning B.J., Geleijnse M.L. (2007). Quantification of left ventricular volumes and function in patients with cardiomyopathies by real-time three-dimensional echocardiography: a head-to-head comparison between two different semiautomated endocardial border detection algorithms. J Am Soc Echocardiogr.

