# Supplementary material for: Changes in Biventricular Cardiac Mechanics After Transcatheter Edge-to-Edge Repair for Severe Tricuspid Regurgitation
Source: JACC Adv. 2026 Jan 21;5(2):102549. doi: 10.1016/j.jacadv.2025.102549 (PMC12859493; doi:10.1016/j.jacadv.2025.102549)
Supplement: Supplemental Figures and Tables [file mmc1.docx]

**SUPPLEMENTAL MATERIAL**

**Changes in biventricular cardiac mechanics after transcatheter edge-to-edge repair for severe tricuspid regurgitation**

**Author**s

Giulio M. Mondellini, MD ^1^*; Antoon J.M. van den Enden, MD ^1^*; Mark M.P. van den Dorpel, MD ^1^; Claire Ben Ren, MD PhD^1^; Christiaan L. Meuwese, MD PhD ^1,2^; Isabella Kardys MD PhD ^1^; Rutger-Jan Nuis, MD PhD ^1^; Maarten ter Horst, MD PhD ^3^; Marcel L. Geleijnse MD PhD ^1^; Joost Daemen MD PhD ^1^; Daniel Burkhoff, MD PhD ^4^; Nicolas M. Van Mieghem, MD PhD ^1^

**Affiliations**

^1^ Department of Cardiology, Cardiovascular Institute, Erasmus University Medical Center, Rotterdam, the Netherlands

^2^ Department of Intensive Care Adults, Erasmus University Medical Center, Rotterdam, the Netherlands;

^3^ Department of Cardiothoracic Anesthesiology, Erasmus University Medical Center, Rotterdam, the Netherlands;

^4^ Cardiovascular Research Foundation, New York City, New York, United States of America.

* Equally contributed

**Brief title**: Effects of Tricuspid TEER on cardiac mechanics

**Corresponding author**

prof. N.M. (Nicolas) Van Mieghem, MD PhD FESC FACC

Professor and Clinical Director of Interventional Cardiology

Erasmus University Medical Center

Dept. of Cardiology, Cardiovascular Institute, office Nt-645

Dr. Molewaterplein 40 3015 GD, Rotterdam, the Netherlands

Phone: +31 10 7035260 - Fax: +31 10 7035254 Email: [n.vanmieghem@erasmusmc.nl](mailto:n.vanmieghem@erasmusmc.nl)

**SUPPLEMENTAL METHODS**

At our institution mechanical ventilation settings, use of vasoactive agents, spasmolytics, and anesthetics adopted during tricuspid TEER procedures are per treating anesthesiologist’s discretion in the best interest of patient. Nevertheless, the anesthesiologists involved in this study adhered to a standardized protocol. All patients received maintenance anesthesia with propofol and remifentanil. The depth of anesthesia was monitored using the bispectral index (BIS). Regarding ventilation setting, a lung-protective ventilation strategy was employed with low tidal volumes (e.g. ≤ 6 ml/kg of ideal body weight) and low positive end-expiratory pressure levels (e.g. 5-10 cmH20, as needed).

In all patients, low-dose norepinephrine was administered and inotropic agents (dobutamine or milrinone) were only used upon anesthesiologist’s discretion.

**SUPPLEMENTAL RESULTS**

We used the Wilcoxon signed-rank test to compare the RV E_es_ values post TEER as measured with the single beat V_0_ vs. the fixed V_0_.

RV E_es_ measured using the single-beat estimated V_0_ significantly increased from 0.46 at baseline (IQR 0.33;1.06) mmHg/mL to 0.91 (IQR 0.73;1.35) mmHg/mL after TEER, (p = 0.010) and this compares to the RV E_es_ post TEER measured using the fixed V_0_ of 0.82 (0.55, 2.07) mmHg/mL. The overall median RV E_es_ value post TEER was similar for both methods, with a median difference of 0.0 mmHg/mL (IQR: -0.38 to 0.21, Wilcoxon signed-rank test, *p* = 0.837), suggesting no significant systematic bias ( Supplemental Table 1).

**SUPPLEMENTAL TABLES**

**Supplemental Table 1**

| Variable, N=21 | RV After TEER  Estimated single-beat V_0_ | RV After TEER  Fixed V_0_ | Difference  RV after TEER  Estimated single-beat V_0_ - Fixed V_0_ |
| --- | --- | --- | --- |
| Ees (mmHg/mL) | 0.91 (0.73;1.35) | 0.82 (0.55,2.07) | 0.0 (-0.38;0.21) |

Variables are presented as median (25^th^,75^th^ percentile) according to normality of the distribution.; *E_es_ = End-Systolic Elastance RV= right ventricular; TEER, transcatheter edge-to-edge repair, V = Volume*

**Supplemental Table 2**

| **N=21** | **RV Ees before TEER** | **RV Ees After TEER**  **Calculated V_0_** | **RV Ees After TEER**  **Fixed V_0_** | **Delta**  **Calculated V_0_ -Fixed V_0_** |
| --- | --- | --- | --- | --- |
| 1 | 0.81 | 1.69 | 1.63 | 0.06 |
| 2 | 1.66 | 0.58 | 1.43 | -0.85 |
| 3 | 0.28 | 0.71 | 0.29 | 0.42 |
| 4 | 0.44 | 1.01 | 0.84 | 0.17 |
| 5 | 0.99 | 0.90 | 3.02 | -2.12 |
| 6 | 0.87 | 1.10 | 1.48 | -0.38 |
| 7 | 4.10 | 6.54 | 6.64 | 0.10 |
| 8 | 2.57 | 1.81 | 2.50 | -0.69 |
| 9 | 0.45 | 0.75 | 0.65 | 0.10 |
| 10 | 0.34 | 1.44 | 0.40 | 1.04 |
| 11 | 0.27 | 0.71 | 0.75 | -0.04 |
| 12 | 0.42 | 0.91 | 0.60 | 0.31 |
| 13 | 0.25 | 0.61 | 0.46 | 0.15 |
| 14 | 1.13 | 0.76 | 3.18 | -2.42 |
| 15 | 0.49 | 0.80 | 0.80 | 0.00 |
| 16 | 0.17 | 0.97 | 0.24 | 0.73 |
| 17 | 0.31 | 0.70 | 0.49 | 0.21 |
| 18 | 0.46 | 1.27 | 0.82 | 0.45 |
| 19 | 0.84 | 1.28 | 3.67 | -2.39 |
| 20 | 0.34 | 0.77 | 0.78 | -0.01 |
| 21 | 1.12 | 1.42 | 1.55 | -0.13 |

Individual values of RV Ees before and after TEER, as measured with the single beat V_0_ vs. the fixed V_0_

**Supplemental Table 3**

| Variables | ≤ Moderate Residual TR  N=15 | | | | | | >Moderate Residual TR  N=7 | | | | | |
| --- | --- | --- | --- | --- | --- | --- | --- | --- | --- | --- | --- | --- |
|  | **RV Before TEER** | **RV After TEER** | **p-value** | **LV Before TEER** | **LV After TEER** | **p-value** | **RV Before TEER** | **RV After TEER** | **p-value** | **LV Before TEER** | **LV After TEER** | **p-value** |
| Heart Rate, bpm | 72.30 ± 17.39 | 63.06 ± 11.79 | 0.016 | 63.44 ± 13.78 | 62.20 ± 14.63 | 0.690 | 66.33 ± 11.45 | 60.99 ± 14.67 | 0.032 | 65.65 ± 14.21 | 59.33 ± 12.64 | 0.044 |
| End-systolic pressure, mmHg | 25.49 ± 9.99 | 35.86 ± 15.41 | <0.001 | 120.47 ± 22.33 | 123.60 ± 17.63 | 0.567 | 22.06 ± 5.80 | 24.70 ± 8.64 | 0.113 | 109.82 ± 17.70 | 100.65 ± 13.50 | 0.102 |
| End-diastolic pressure, mmHg | 8.56 ± 3.81 | 7.62 ± 4.05 | 0.363 | 14.20 ± 3.57 | 15.53 ± 4.68 | 0.220 | 6.26 ± 3.51 | 4.91 ± 3.55 | 0.211 | 11.88 ± 3.62 | 11.92 ± 3.86 | 0.973 |
| End-systolic volume, mL | 68.50 ± 22.10 | 64.59 ± 23.31 | 0.058 | 64.58 ± 25.81 | 68.57 ± 24.79 | 0.097 | 63.54 ± 12.92 | 57.80 ± 8.94 | 0.030 | 70.06 ± 26.32 | 76.33 ± 28.20 | 0.024 |
| End-diastolic volume, mL | 118.44 ± 35.57 | 105.43 ± 30.30 | <0.001 | 104.70 ± 31.15 | 110.31 ± 30.41 | 0.013 | 107.63 ± 24.91 | 95.27 ± 18.12 | 0.065 | 114.06 ± 34.70 | 120.71 ± 36.64 | 0.037 |
| Stroke volume, mL | 46.36 ± 20.05 | 35.71 ± 15.31 | 0.027 | 37.30 ± 8.63 | 37.27 ± 13.45 | 0.994 | 35.95 ± 12.40 | 40.66 ± 17.05 | 0.328 | 40.04 ± 12.50 | 41.11 ± 14.32 | 0.575 |
| dP/dt _max_, mmHg/sec | 185.48 ± 96.85 | 240.44 ± 107.38 | 0.014 | 1098.18 ± 219.55 | 1036.91 ± 208.47 | 0.089 | 201.79 ± 74.59 | 219.37 ± 64.17 | 0.070 | 914.00 ± 194.64 | 811.23 ± 148.08 | 0.081 |
| E_es_ (End-systolic elastance), mmHg/mL | 0.47 [0.33–1.12] | 0.81 [0.57–3.06] | 0.003 | 1.84 [1.32–2.36] | 1.86 [1.26–2.57] | 0.650 | 0.45 [0.28–0.87] | 0.84 [0.46–1.63] | 0.043 | 1.09 [1.05–2.00] | 1.03 [0.92–1.55] | 0.075 |
| V_120_ , mL | 239.97 [151.18–386.81] | 166.72 [124.97–193.91] | 0.008 | 73.91 [39.17–91.11] | 76.13 [57.25–91.85] | 0.311 | 275.04 [166.46–461.26] | 155.68 [128.95–199.63] | 0.028 | 84.04 [63.88–98.54] | 93.63 [77.41–125.93] | 0.176 |
| E_a_, (Arterial elastance), mmHg/mL | 0.54 [0.41–0.84] | 1.02 [0.67–1.36] | 0.001 | 3.53 [2.41–4.08] | 3.43 [2.51–4.73] | 0.345 | 0.57 [0.52–0.62] | 0.66 [0.44–1.08] | 0.396 | 2.72 [1.94–4.49] | 2.36 [1.83–3.89] | 0.128 |
| E_es_/E_a_ | 0.99 [0.66–2.13] | 1.21 [0.51–3.83] | 0.379 | 0.51 [0.47–0.83] | 0.59 [0.30–0.81] | 0.917 | 0.78 [0.56–1.59] | 1.40 [1.04–2.20] | 0.046 | 0.45 [0.39–0.54] | 0.47 [0.37–0.55] | 0.865 |
| Stroke Work, mmHg/mL | 796.48 [600.83–1155.22] | 796.85 [617.62–1678.65] | 0.124 | 3344.70 [2918.20–4977.90] | 4269.80 [2494.00–5288.45] | 0.917 | 952.17 [467.30–1342.50] | 906.00 [444.50–1512.00] | 0.176 | 3528.40 [2438.80–4434.70] | 3968.60 [3092.10–4552.70] | 0.499 |
| Potential Energy, mmHg/mL | 640.47 [154.59–881.87] | 673.97 [100.62–1535.55] | 0.158 | 4058.81 [2239.40–5467.05] | 3811.77 [2887.81–5280.37] | 0.600 | 426.34 [328.57–746.93] | 323.15 [208.62–352.46] | 0.043 | 3498.48 [3070.88–6185.20] | 4400.72 [3787.47–5118.91] | 0.499 |
| Pressure Volume Area, mmHg/mL | 1380.81 [821.55–2272.15] | 1702.20 [736.46–2608.18] | 0.198 | 7949.24 [5569.49–9223.37] | 8081.57 [6410.94–10541.17] | 0.861 | 1471.92 [795.87–1768.84] | 1137.04 [767.65–1864.46] | 0.499 | 7094.10 [6599.28–9223.37] | 8644.52 [7243.60–9087.51] | 0.612 |
| SW / PVA ratio | 0.61 ± 0.17 | 0.64 ± 0.20 | 0.418 | 0.50 ± 0.11 | 0.48 ± 0.15 | 0.447 | 0.66 ± 0.10 | 0.73 ± 0.09 | 0.093 | 0.43 ± 0.05 | 0.47 ± 0.06 | 0.286 |
| dP/dV, mmHg/mL | 24.16 [16.87–30.62] | 22.68 [13.02-27.39] | 0.221 | 43.68 [34.34–49.97] | 48.04 [33.53–72.23] | 0.382 | 15.60 [10.02–29.27] | 11.48 [4.16 – 20.70] | 0.176 | 29.99 [28.10–53.46] | 31.27 [27.47–50.31] | 0.345 |
| -dP/dt min, mmHg/sec | -162.20 [-237.03;-128.44] | -275.15 [-425.27;-205.97] | 0.011 | -1227.70 [-1305.20;-1012.10] | -1179.00 [-1284.60;-1008.38] | 0.600 | -165.00 [-214.40;-97.00] | -169.40 [-227.00;-153.50] | 0.128 | -909.20 [-995.80;-892.30] | -950.40 [-985.20;-768.30] | 0.499 |
| Tau, ms | 59.67 [43.75-145.65] | 43.60 [32.75-55.03] | 0.009 | 39.20 [35.20-44.00] | 37.30 [36-47] | 0.326 | 50.67[33.60-57.30] | 38.30 [26.67-52.70 | 0.499 | 37.71 [37.00-47.30] | 42.30[38.50-46.40] | 0.310 |

*Variables are presented as mean ± standard deviation (SD) or median [25th – 75th percentiles]*

Comparisons of RV and LV mechanics before and after the tricuspid TEER within each subgroup were performed using paired samples t-test for normally distributed variables or Wilcoxon signed-rank test for non-normal distributions.

*dP/dtmax = maximum rate of pressure change over time; Ea = Arterial Elastance; Ees = End-Systolic Elastance; ESP = End-Systolic Pressure; ESV = End-Systolic Volume; LV, left ventricular; RV, right ventricular; PVA = Pressure Volume Area; SW = Stroke Work; TEER, transcatheter edge-to-edge repair, V = Volume*

**SUPPLEMENTAL FIGURES**

**Supplemental Figure 1.**

**
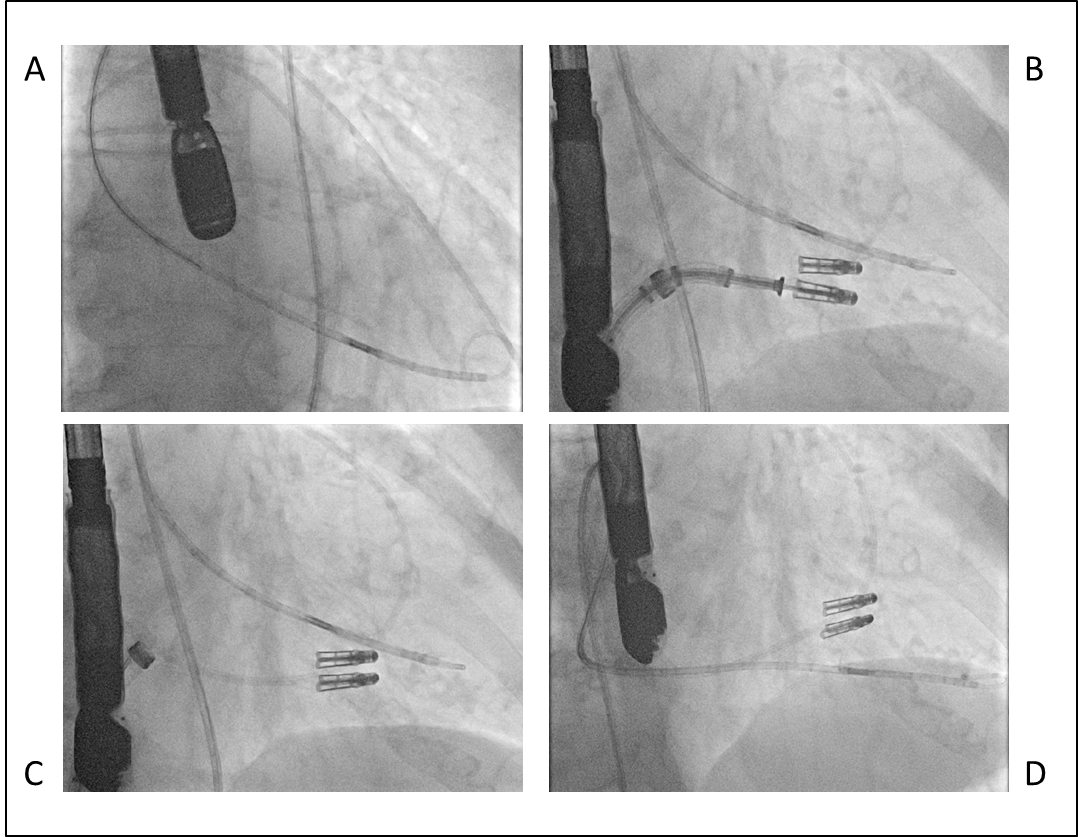
**

Biventricular Pressure-Volume (PV) reconstructions were based on conductance catheter measurements in-vivo before and after tricuspid Transcatheter Edge-to-Edge Repair (TEER).

A = Conductance catheter in the left ventricle (LV) before TEER. B= The conductance catheter remained in the LV during the TEER procedure. C= LV PV measurements were repeated after TEER. D = RV PV measurements after TEER.

**Supplemental Figure 2.**

**
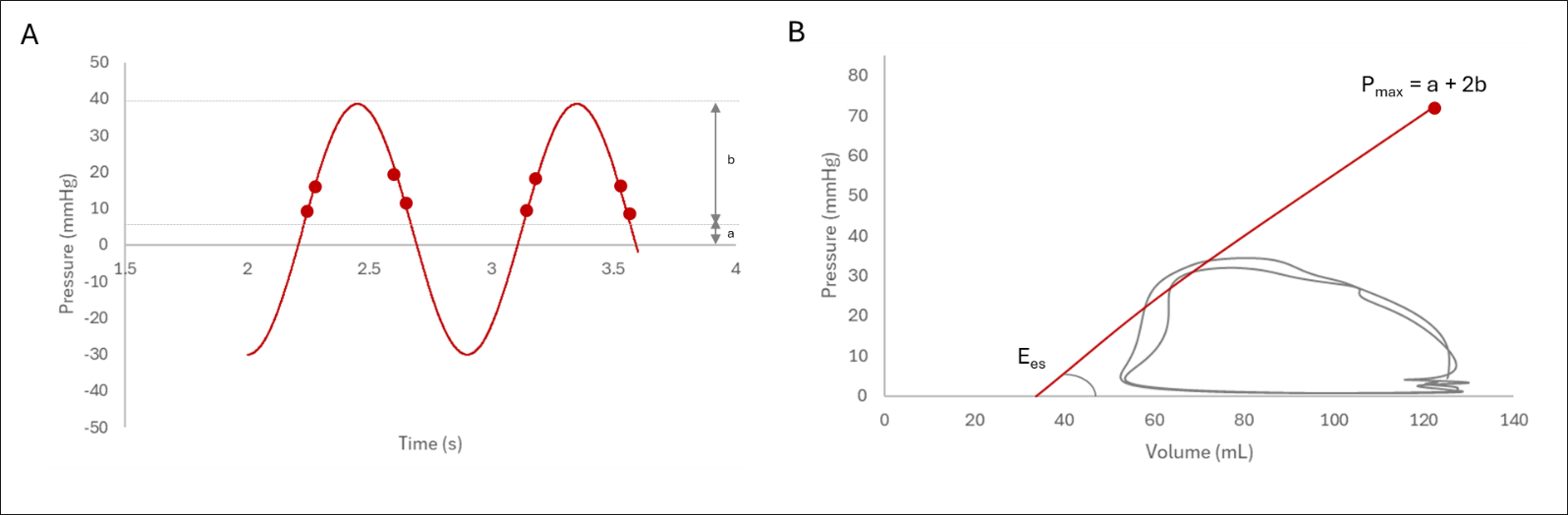
**

Panel A: Example of a sinusoidal curve fitted through the identified key pressure/time points used to define fitting ranges on the right ventricular pressure waveform, using the equation P =a + b · sin (c · t +d), to determine coefficients a-d, through the equation P =a + b · sin (c · t +d).

Panel B: Graphic illustration of RV end-systolic elastance (E_es_) single-beat estimation, Pmax was calculated as Pmax = a + 2b. RV E_es_ represented as the slope of the ESPVR line.

**Supplemental Figure 3**


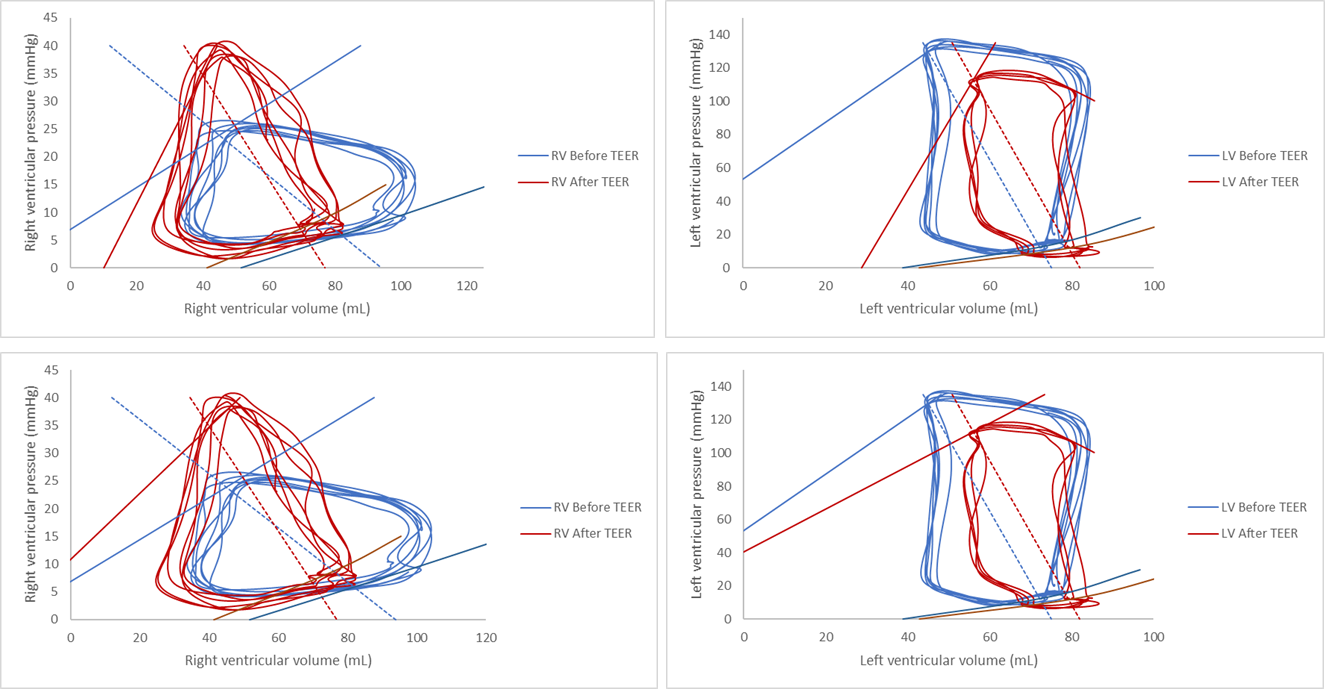


Examples of biventricular pressure-volume reconstructions before and after TEER for severe tricuspid regurgitation.

Outliers in measured V_0_  may hamper the Ees interpretation (see top panels, in which RV and LV Ees after TEER with calculated V_0mmHg_ were 0.91 and 3.44 mmHg/mL, respectively). We therefore also calculated E_es_ after TEER with fixed V_0mmHg_ (i.e. the V_0_ before TEER) as ESP/[ESV – V_0_], where ESP is end-systolic pressure and ESV is end-systolic volume, as illustrated in the lower panels, (in which RV and LV Ees after TEER with fixed V_0mmHg_ were 0.60 and 1.29 mmHg/mL, respectively).

*^a^ V_0mmHg_ = volume-axis intersection of the end-systolic pressure-volume relation trendline with slope E_es_. LV = Left Ventricular; RV = right ventricular TEER = Transcatheter edge-to-edge repair*

**Supplemental Figure 4.**

A flow-chart enrolled patients, dropouts and respective causes

**
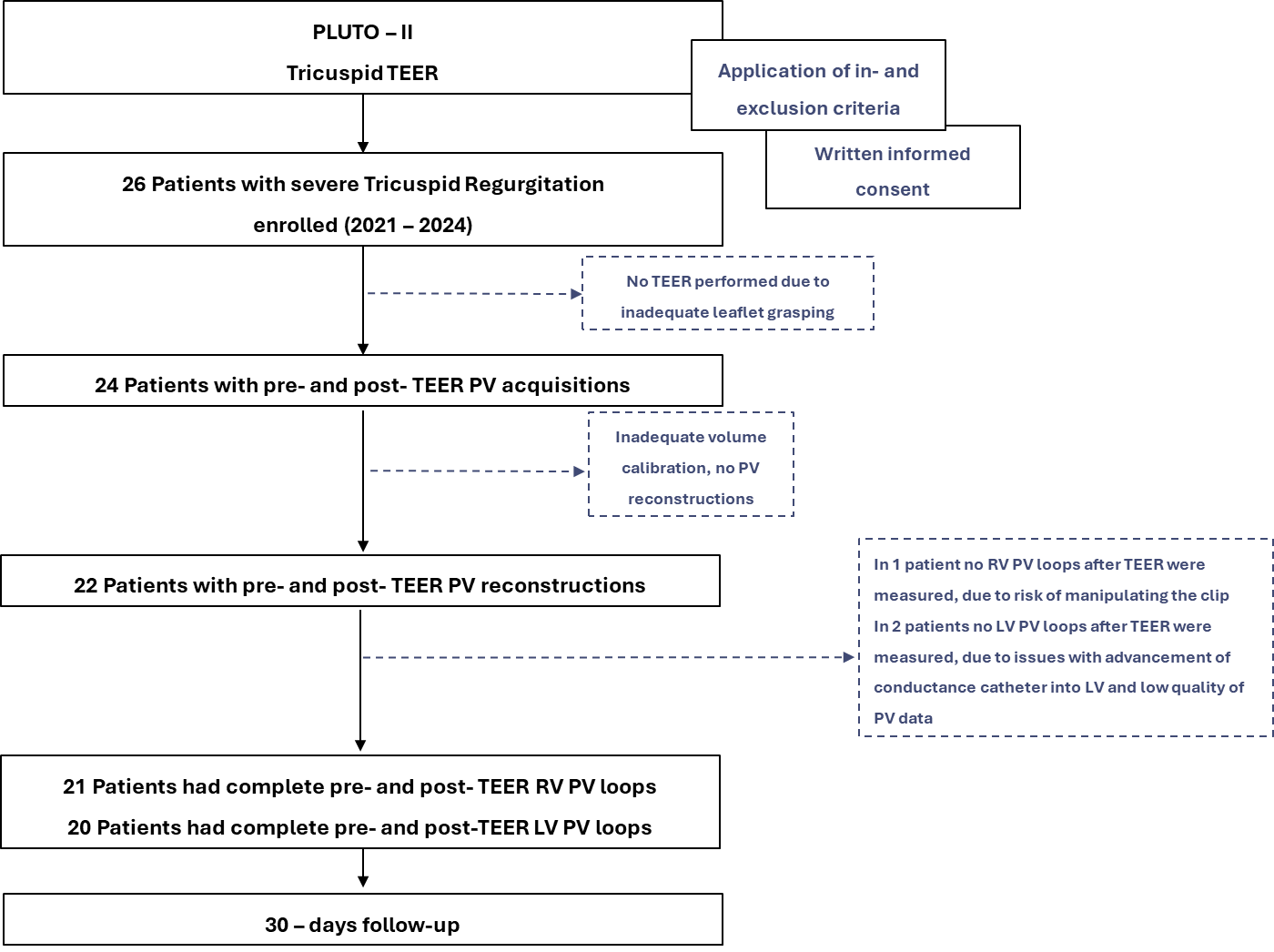
**

**Supplemental Figure 5.**

Visual representation of changes in RV contractility, indexed by E_es_, with paired dot plots before and after tricuspid TEER

**
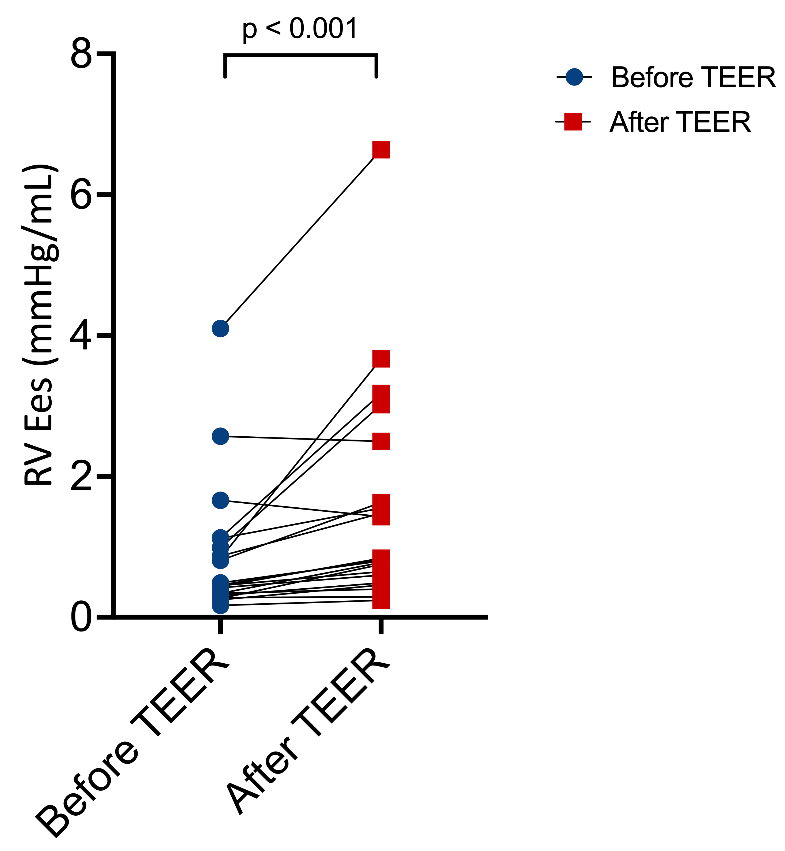
**

**Supplemental Figure 6.**

**
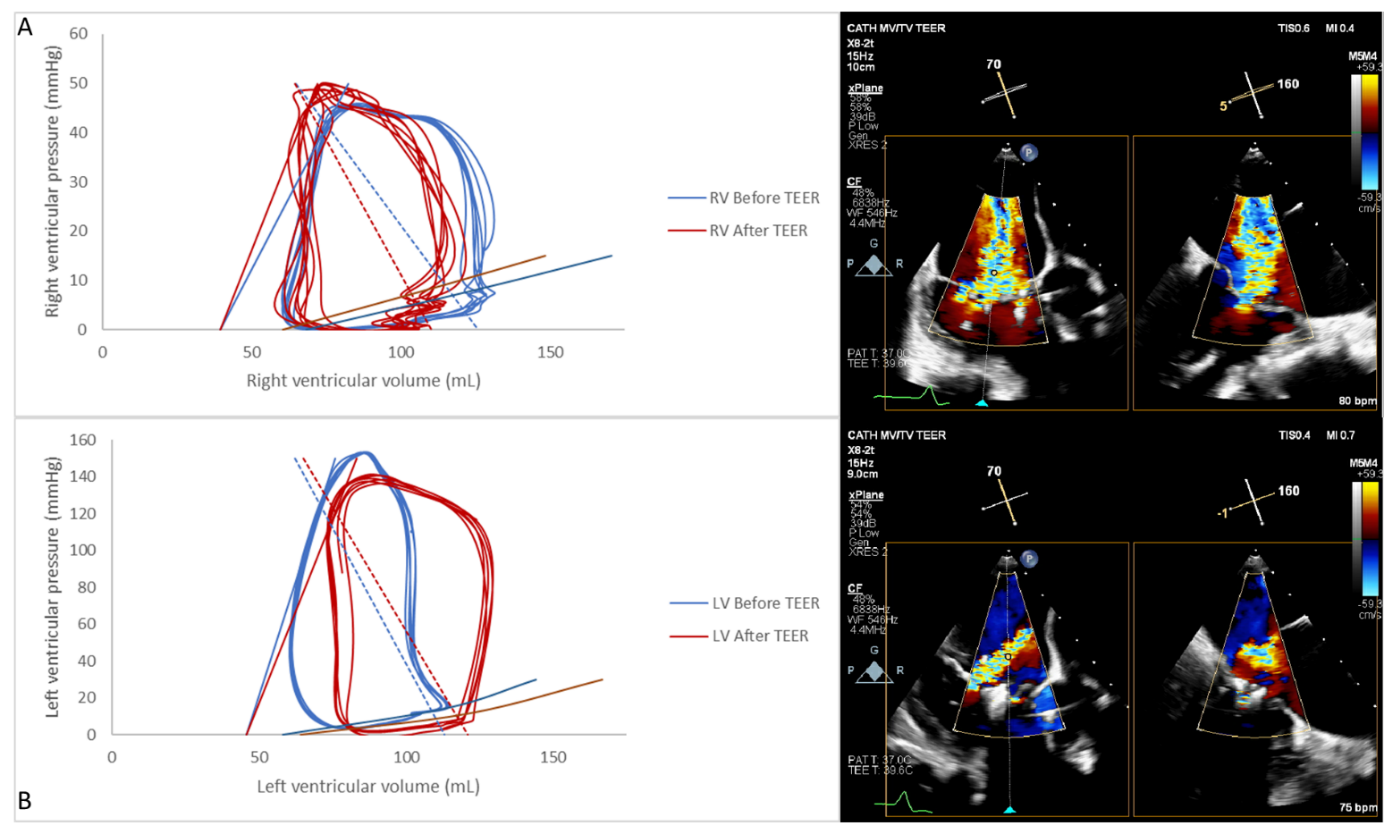
**

Right (A) and left (B) ventricle PV loops, before and after tricuspid Transcatheter Edge-to-Edge Repair (TEER), in a 65-year-old-male patient with massive secondary TR with tethering of anterior and posterior leaflets, impaired RV systolic function and dual-chamber pacemaker. Two TriClip (Abbott) were positioned, achieving mild residual TR.

Right ventricular PV loops before and after TEER (A), show a reduction of end-diastolic volume, an increase in effective arterial elastance (E_a_) and in end-systolic elastance (E_es_) a load-independent measure of contractility after the procedure (in red).

Left ventricular PV loops before and after TEER (B) show the increase of end-diastolic volume after the procedure.

Color-Doppler imaging in transesophageal echocardiography before (top right panel) and after (bottom right panel) tricuspid TEER.

**Supplemental Figure 7.**

**
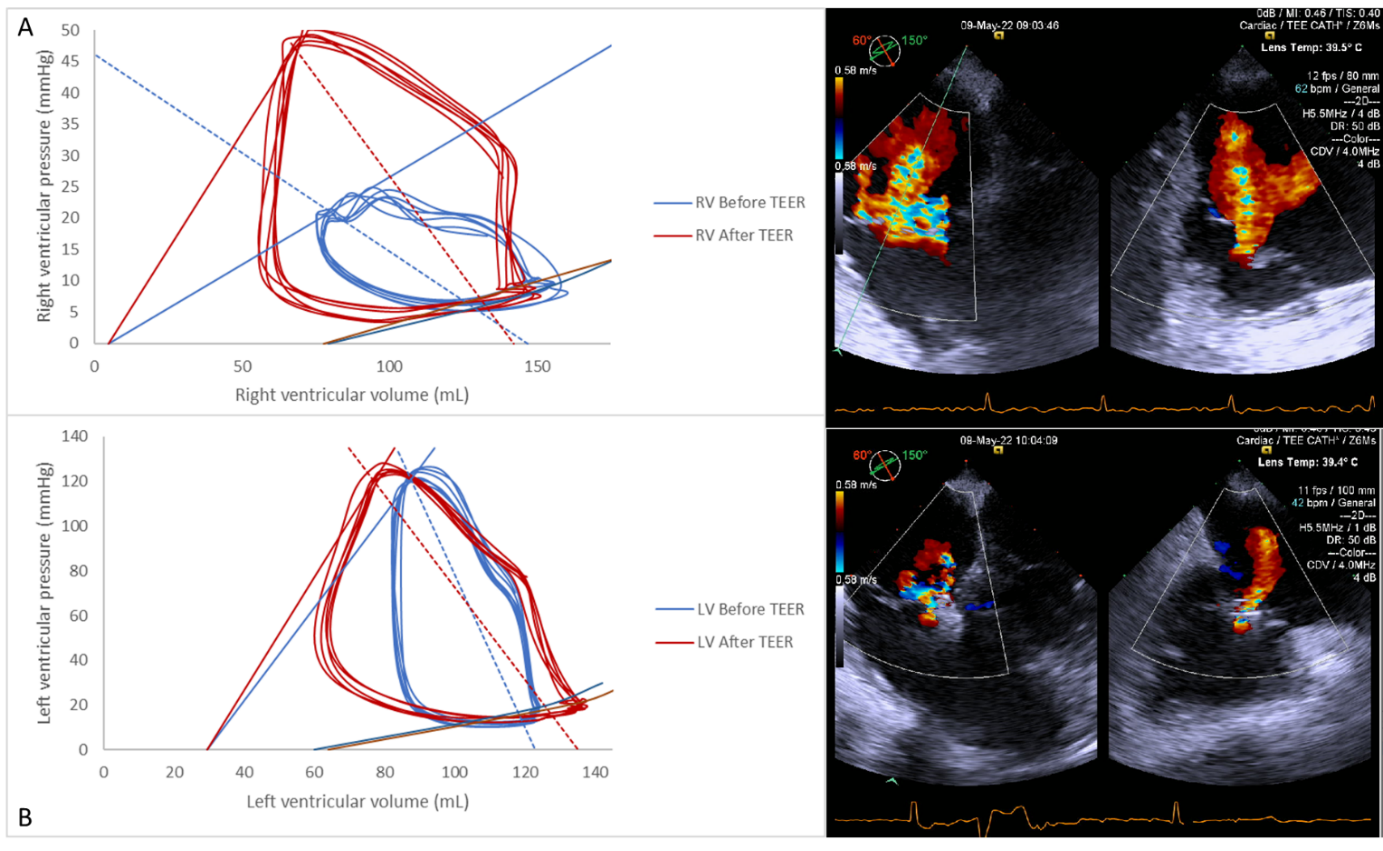
**

Right (A) and left (B) ventricle PV loops, before and after tricuspid Transcatheter Edge-to-Edge Repair (TEER), in 80-year-old-female patient with severe TR and severe bi-atrial enlargement. One TriClip (Abbott) was positioned achieving mild residual TR.

Right ventricular PV loops before and after TEER (A), show a reduction of end-diastolic volume, an increase in effective arterial elastance (E_a_) and in end-systolic elastance (E_es_) a load-independent measure of contractility after the procedure (in red). Left ventricular PV loops (B) show the increase of end-diastolic volume after TEER.

Color-Doppler imaging in transesophageal echocardiography before (top right panel) and after (bottom right panel) TEER.
